# Supplementary material for: Getting More Out of Biomedical Documents with GATE's Full Lifecycle Open Source Text Analytics
Source: PLoS Comput Biol. 2013 Feb 7;9(2):e1002854. doi: 10.1371/journal.pcbi.1002854 (PMC3567135; doi:10.1371/journal.pcbi.1002854)
Supplement: Dataset S1 — GATE software. Dataset S1 bundled with this paper contains a distribution of GATE (or see http://gate.ac.uk/download/). (TGZ) [file pcbi.1002854.s001.tgz › plos-gate/src/gate/resources/gate.ac.uk/tests/gu-Am-Brit-4-aug-2001.html]

Guardian Unlimited | Archive Search


|  |  |
| --- | --- |
|  |  |


|  |  |
| --- | --- |
|  | **Go to:**  Guardian Unlimited home Politics Books EducationGuardian.co.uk Clearing Film Sport Football Jobs MediaGuardian.co.uk SocietyGuardian.co.uk Money Shopping Travel The Observer Work Search the archive ---------------------- News Audio Net news Special reports The Guardian UK latest The weblog The newslist The wrap World latest Arts latest ---------------------- Crossword Content distribution Events / offers Help / contacts Information Life online Notes & Queries Style guide Travel offers TV listings Weather Web guides ---------------------- Guardian Weekly Money Observer |


|  |  |  |  |  |  |  |  |  |  |  |  |  |  |  |  |  |  |  |  |  |  |  |  |  |  |  |  |  |  |  |  |  |  |  |  |  |  |  |
| --- | --- | --- | --- | --- | --- | --- | --- | --- | --- | --- | --- | --- | --- | --- | --- | --- | --- | --- | --- | --- | --- | --- | --- | --- | --- | --- | --- | --- | --- | --- | --- | --- | --- | --- | --- | --- | --- | --- |
| Network home |  |  |  |  | UK news |  |  |  |  | World latest |  |  |  |  | Books |  |  |  |  | Money |  |  |  |  | Film |  |  |  |  | Society |  |  |  |  | The Observer |  |  |  |
|  | | | | | | | | | | | | | | | | | | | | | | | | | | | | | | | | | | | | | | |
| Politics |  |  |  |  | Education |  |  |  |  | Shopping |  |  |  |  | Work |  |  |  |  | Football |  |  |  |  | Jobs |  |  |  |  | Media |  |  |  |  | Search |  |  |  |


|  |  |  |
| --- | --- | --- |
|  |  |  |
|  |  | Notebook **Am-Brit Airways might just manage to fly**  Green light likely for joint venture  **Guardian** **Saturday August 4, 2001** It is five years since British Airways first attempted to cook up a cooperation deal with American Airlines. That approach came to nought when the rock of the competition authorites met the hard place of British Airways' refusal to give up enough valuable slots at Heathrow to get the deal through. The negotiations were seemingly interminable, cost a fortune, distracted management and eventually contributed to the demise of BA's then chief executive, Bob Ayling. But yesterday the two parties announced they were trying again, and this time the deal is different. Last time it was all about selling tickets on one another's planes. This time it is a "profit-sharing" joint venture, which will allow them to put one big, economical plane on a route instead of two smaller, less efficient ones. They are planning to work together on just nine routes, instead of the 22 they had aimed for last time. Since the last deal was mooted, competition has altered on the transatlantic routes, with British Midland joining the Star Alliance headed by Lufthansa and United Airlines. The savings for BA from the new deal would be enormous - yesterday one analyst hastily calculated they could be 15% on each route. The sticking point will again be slots at Heathrow. Last time the EU wanted the airlines to surrender 267 a week. This time, with the changes in competition, it could be half that and BA may be allowed to sell them rather than give them away. The two airlines would not have come back with a new plan without some inkling from the competition watchdogs that their flight path might now be cleared for take-off. This time, it might just fly. **First, dig a hole**   Who cares about the imbalances, go for growth. That seems to be the message on both sides of the Atlantic these days. In Britain, the Bank of England has sent a clear signal that if the only way to prop up the economy is a consumer and housing market boom, then so be it. In the US, policymakers are even less subtle. The Internal Revenue Service website offers some handy tips for households wondering what to do with the $300 tax rebate cheques they received this week: "Let's see" it says. "New barbecue, early holiday shopping . . ." There are two schools of thought on whether the consumer can rescue the world economy from the mess it seems to have got itself into. One advocates throwing caution to the wind, with interest rate cuts and fiscal boosts. The other worries that the imbalances in US and British economies will have to be corrected, and they may have a point. Both are running large current account deficits and their personal sectors are increasingly dipping into the red. At some stage the rest of the world will decide it no longer wants to finance the British or US consumption habit. On this occasion, though, we find ourselves in sympathy with the "shop till you drop" approach. It seems like good Keynesian common sense. With growth faltering, both economies need an old-fashioned boost to demand. The Fed and the Bank have both responded with lower borrowing costs. The boost to public spending in Britain together with tax rebates in the US should add some more momentum. If there were any signs of inflationary pressures, the Bank and the Fed are capable of reacting quickly. In the meantime, it is time to indulge in a little retail therapy for the sake of the economy - and prove we can spend our way out of recession. **Unileveraged**   Looming recession did not prevent Unilever from sticking to its forecasts of double-digit earnings growth for this year. The food and personal care group is in the throes of a vast restructuring - which goes by the title of "Path to Growth". The plan, revealed with much hullabaloo last year, is to focus Unilever's considerable resources behind only power brands, so it is axing or selling 1,200 of the 1,600 brands it owned this time last year. Out have gone dowdy old products like Batchelors dried peas, to allow Unilever to put more oomph behind winners like Dove soap and Magnum ice creams. At the same time it has been buying labels it can leverage worldwide - like Hellmans mayonnaise, the French Amora mustard business, diet product Slimfast and Ben & Jerry's right-on ice cream parlor. The fallout from this overhaul is 125 redundant factories and a 30,000 cut in its payroll. So far it all seems to be going according to plan - 700 brands have gone so far. In a year the shares have climbed from 340p to more than 600p. If recession should bite, Unilever will be better placed than many to ride it out - its core food, personal care and cleaning products will be the last to feel the impact of any prudent consumer belt-tightening. The shares are a firm hold. |
|  |  |  |


|  |  |  |  |  |
| --- | --- | --- | --- | --- |
|  | Guardian Unlimited © Guardian Newspapers Limited 2001 | | | |
